# Virucidal activity of oriental hornet *Vespa orientalis* venom against hepatitis C virus

**DOI:** 10.1590/1678-9199-JVATITD-2021-0039

**Published:** 2021-11-19

**Authors:** Moustafa Sarhan, Alaa M. H. El-Bitar, Amaal Mohammadein, Mohammed Elshehaby, Hak Hotta

**Affiliations:** 1 Molecular biology Laboratory, Zoology Department, Faculty of Science, Al-Azhar University, Assiut, Egypt.; 2 Department of Microbiology, Kobe University Graduate School of Medicine, Kobe, Japan.; 3 Zoology Department, Faculty of Science, Al-Azhar University, Assiut, Egypt.; 4 Zoology Department, Faculty of Science, Zagazig University, Zagazig, Egypt.; 5 Department of Biology, College of Science, Taeif University, Saudi Arabia.; 6 Faculty of Clinical Nutrition and Dietetics, Konan Women’s University, Kobe, Japan.

**Keywords:** Wasp venom, Vespa orientalis, Hepatitis C virus (HCV), Antiviral activity

## Abstract

**Background:**

Hepatitis C virus (HCV) infection is a major worldwide health problem that can cause liver fibrosis and hepatocellular carcinoma (HCC). The clinical treatment of HCV infection mainly relies on the use of direct-acting antivirals (DAAs) that are usually expensive and have side effects. Therefore, achieving the discovery of more successful agents is always urgent. In this context, antiviral compounds that inhibit viral infections and disease progression with important therapeutic activities have been identified in animal venoms including arthropod toxins. This indicates that arthropod venoms represent a good natural source of promising candidates for new antivirals.

**Methods:**

The antiviral activity of the wasp venom (WV), isolated from the Oriental hornet (*Vespa orientalis*), was assessed using cell culture technique with human hepatocellular carcinoma-derived cell line (Huh7it-1) and the recombinant strain of HCV genotype 2a (JFH1).

**Results:**

The results revealed that WV inhibited HCV infectivity with 50% inhibitory concentration (IC_50_) of 10 ng/mL, while the 50% cytotoxic concentration (CC_50_) was 11,000 ng/mL. Time of addition experiment showed that the WV blocked HCV attachment/entry to the cells probably through virucidal effect. On the other hand, the venom showed no inhibitory effect on HCV replication.

**Conclusion:**

WV can inhibit the entry stage of HCV infection at non-cytotoxic concentrations. Therefore, it could be considered a potential candidate for characterization of natural anti-HCV agents targeting the entry step.

## Background

The Arthropoda constitute the largest phylum in the animal kingdom, which has evolved and diversified to include millions of species. The success of the arthropods’ flourishment is due most probably to the variation of their venom apparatus and venom usage [1,2]. Thousands of arthropod species are venomous and utilize their venoms to defend, predate, and paralyze prey as arachnids (scorpions and spiders) and hymenopterans (wasps, ants, and bees). Arthropod venoms such as those from scorpions, spiders and wasps are sometimes harmful to humans and may cause serious injuries. At the same time, those venoms are potentially useful biopharmaceuticals and biotools. For these reasons, venomous animals have attracted the attention as a rich source for seeking to characterize novel biologically active compounds. 

Oriental hornet (*Vespa orientalis;* Linnaeus, 1771) is a social insect of the family Vespidae that is found in north Africa including Egypt. *V. orientalis*uses its venom for feeding or for defense purposes. This venom has several physical and pharmacological properties due to the nature of its chemical composition. Stings by*V. orientalis* produce serious systemic symptoms such as severe pain and local irritation progressing to urticarial papules. Recently, the studies of bioactive compounds of wasp venom have attracted many researchers; these compounds include small peptides, amines, and low/high molecular weight proteins such as enzymes and toxins [3]. 

Wasp venom (WV) constituents include serotonin, acetylcholine, kinins, adrenaline, noradrenaline, dopamine, and high molecular weight compounds such as hyaluronidase, phospholipase-A_2_, histidine decarboxylase, poly and disaccharidase, neutral DNase, and several polycationic peptides that act together to give the biological effects of this venom [4,5]. However, the antiviral features of WV have not been well examined and its mode of action is not completely understood. 

HCV causes serious health problems worldwide, with no effective vaccine available. HCV infection is a major cause of HCC [6-8]. Recently, the U. S. Food and Drug Administration (FDA) approved DAAs for HCV treatment, but shortly after HCV eradication with DAA, an observed increase in early occurrence or recurrence of HCC has been reported [9-11] which led to a lot of studies with contradictory results [12]. Moreover, the DAAs have the disadvantages of gene selectivity, resistance and low accessibility to mutated virus strains, the risk of sustained immune response and expensive cost [13]. For these reasons, the development of novel and safe compounds with improved efficacy is still required for HCV treatment. In the present study, the antiviral effects of WV on the HCV replication life cycle were evaluated *in vitro*.

## Methods

### Wasp venom collection

Live wasps were captured in the area of Al Wilidiyyah, Assiut province, Egypt and kept at -20°C. Vespid venoms were collected by removing the venom sacs from fresh frozen insects and squeezed out into buffer solution. Collected venoms were lyophilized, dissolved in distilled water, aliquoted, and stored at -20ºC.

### Cell culture and viruses

Huh7it-1 cells [14], was grown in Dulbecco's modified Eagle's medium (DMEM; Wako, Japan) supplemented with 100 IU/mL penicillin (Invitrogen), 100 μg/mL streptomycin (Invitrogen), non-essential amino acids (Invitrogen, USA), and 10% fetal bovine serum (FBS; Gibco). The cells were maintained at 37°C in a 5% CO_2_ incubator. The supernatants from Huh7it-1 cells infected with cell culture-adapted HCV (JFH1 strain of genotype 2a) [15,16] were collected and stored at ˗80°C until being used. 

### WST-1 assay

Water soluble tetrazolium salts (WST-1) assay was performed for cytotoxicity check as described [7]. In brief, Huh7it-1 cells seeded in a 96-well plate were treated with serial dilutions (2.5 to 40 µg/mL) of crude WV or complete medium as a control for 48 h at 37°C. WST-1 reagent (Roche, Mannheim, Germany) was added to the cells and incubated for 4 h. The number of living cells in each well was determined using a microplate reader. The absorbance was measured at 562 nm. Percent cell viability compared to the control was calculated for each dilution of the WV and the CC_50_ values were determined by SPSS probit analysis in SPSS software (SPSS Inc., Chicago, IL). 

### Analysis of antiviral activities of crude wasp venom

Huh7it-1 cells in 24-well plates were incubated for 2 h with a mixture of fixed amount of HCV and serial dilutions of crude venom (0.5 to 5,000 ng/mL). After washing of cells, a fresh medium containing the same concentrations of the crude venom as those used during virus inoculation was added to the cells. Culture supernatants were harvested at 1 and 2 days post-infection (dpi) and titrated for virus infectivity. Percent inhibition of the virus infectivity by each concentration of WV was calculated by comparing to the control by using SPSS probit analysis in SPSS software, and IC_50_ values were determined. 

### Determination of viral yield in cell culture supernatant (virus titration)

Monolayers of Huh7it-1 cells grown on coverslips in 24-well plates were incubated with culture supernatants of virus-infected cells. After 2 h adsorption at 37ºC, the plates were washed and re-incubated with fresh medium for 22 h. Cells were washed with phosphate-buffered saline (PBS), fixed with 4% paraformaldehyde, and permeabilized with 0.1% Triton X-100 in PBS at room temperature. The cells were incubated with heat-inactivated patient’s serum containing anti-HCV antibody for 1 h, followed by incubation with FITC-conjugated goat anti-human IgG (Medical & Biological Laboratories Co., Ltd., Japan). The cells were counterstained with Hoechst 33342 (Molecular Probes, Eugene, OR, USA) for 5 min. The coverslips were mounted on a glass slide with Vectashield H-1000 reagent (Vector Laboratories, Inc. Burlingame, CA, USA) and HCV-infected cells were counted under a BZ-9000 fluorescence microscope (Keyence, Osaka, Japan).

### Addition of wasp venom at different steps of virus life cycle (time-of-addition experiments)

Huh7it-1 cells were seeded at 5 × 10^5^ cells/well in 6-well plates. The crude WV was added to the cells at a final concentration of 500 ng/mL during different times of infection with HCV at 0.5 m.o.i., as follows: (a) cells were pre-incubated with the WV for 2 h (pre-treatment cells); (b) the virus was pre-incubated with the WV for 2 h, and then the mixture was used for infection (pre-treatment virus); (c) the WV was added only during viral adsorption period for 2 h (treatment during adsorption); (d) the WV was added instantly after the adsorption period (treatment post-infection). (e) the WV was added during and after the adsorption periods (treatment during and after infection). After 2 h incubation (at 37°C) as indicated above, cells were washed and replaced with WV-free medium, whereas WV remained in the medium in experiments (d) and (e). Supernatants were collected at 1 and 2 dpi and titrated for virus infectivity. Also, the cells were subjected to immunoblot analysis for intracellular accumulation of viral proteins at 1 and 2 dpi. 

### Immunoblot analysis

The treated cells were mixed well in 50 µL SDS sample buffer, collected separately in Eppendorf tubes and kept at ˗80°C until use. Proteins of the cell lysates were separated by SDS-polyacrylamide gel electrophoresis (SDS-PAGE) and transferred onto a polyvinylidene difluoride membrane. Target proteins were probed with the indicated primary and horse-radish peroxidase (HRP)-conjugated secondary antibodies as previously described by Sarhan et al. [8]. Detected protein bands were analyzed using the Fujifilm LAS-4000 luminescent image analyzer (ECL; GE Healthcare, Buckinghamshire, UK).

### Real-time quantitative RT-PCR

The NS5A regions of the HCV genome were amplified by reverse transcription-quantitative polymerase chain reaction (RT-qPCR) in a MicroAmp 96-well reaction plate using the ABI PRISM 7500 fast system (Applied Biosystems, Foster City, CA, USA) with SYBR Premix ExTaq (Takara Bio, Kyoto, Japan) as previously described [6]. Human glyceraldehyde 3-phosphate dehydrogenase (GAPDH) mRNA served as an internal control

### Statistical analysis

Data are presented as mean ± SEM (standard error of mean) obtained from at least 2 independent experiments and Student’s *t-*test was used to calculate the statistical significance. *P* <0.05 was considered significant.

## Results

### Analysis of the inhibitory effect of wasp venom against hepatitis C virus

**Figure 1.**
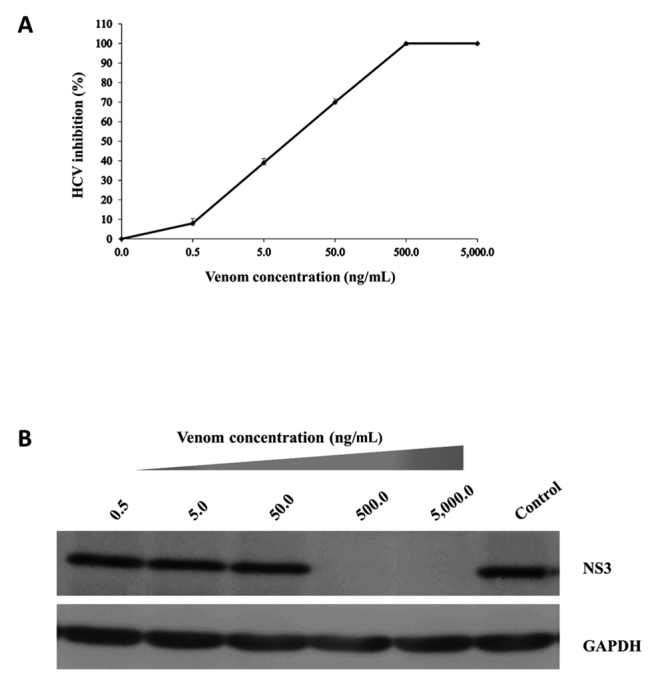
The anti-HCV activity of WV is dose dependent. Serial dilutions of WV were mixed with a fixed amount of HCV and inoculated to Huh7it-1 cells at a multiplicity of infection of 0.5 pfu/cell. The cells were washed and re-incubated in medium containing the same concentrations of WV for 46 h. **(A)** Amount of HCV infectious particles in the supernatants. The culture supernatants were collected and checked for virus infectivity. Percent of HCV inhibition at different concentrations (0.5 to 5,000 ng/mL) was shown. Data were obtained from two independent experiments and represented as means ± SEM. **(B)** Accumulation of HCV NS3 protein inside the cells. Virus-infected cells were analyzed by immunoblot using an anti-HCV NS3 antibody. GAPDH was used as an internal control.

To check whether WV has an anti-HCV activity, serial dilutions of WV (0.5 to 5,000 ng/mL) were mixed with a fixed amount of HCV viral particles and then inoculated to Huh7it-1 cells. The culture supernatants at 1 and 2 dpi were titrated for virus infectivity. Results showed that WV inhibited HCV with an IC_50_ of 10.0 ng/mL and a signiﬁcant dose-dependent manner of anti-HCV activity of WV was observed (Figure 1A and 1B). Also, the data demonstrated that WV exhibited CC_50_ of 11,000 ng/mL and a selectivity index (SI) of 1,100 (Table 1).

**Table 1.** Determination of IC_50_ and CC_50_ of wasp venom against hepatitis C virus.

Venom	IC_50_ (ng/mL)^a^	CC_50_ (ng/mL)^b^	SI^c^
WV (*Vespa orientalis*)	**10.0**	**11,000**	**1,100**

^a^ IC_50_: 50% inhibitory concentration; ^b^CC_50_; 50% cytotoxic concentration; ^c^SI: selectivity index (CC_50_/IC_50_).

### Analysis of the inhibitory effect of wasp venom against hepatitis C virus added at different times of viral infection (time-of-addition experiments)

**Figure 2.**
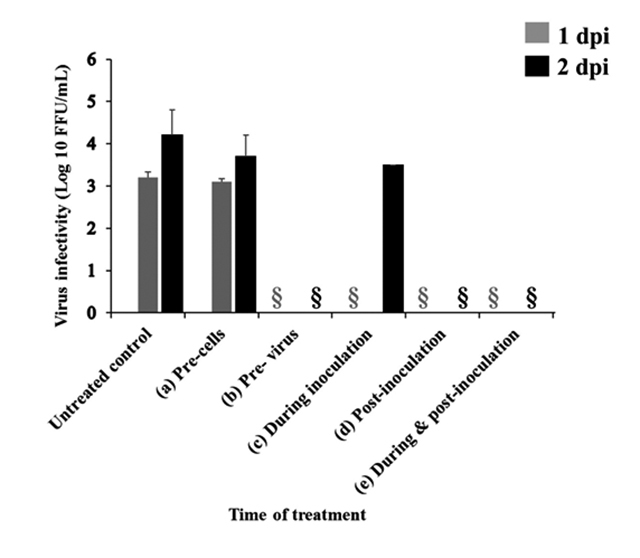
Effect of the addition of WV at various time points during HCV infection. Huh7it-1 cells and/or HCV were treated with WV (500 ng/mL) at various time points and HCV production in the culture was determined. **(a-e)** Quantity of HCV particles in the supernatants. The intracellular virus in each set experiment was quantified 48 hours after infection by analyzing focus forming units per milliliters (FFU/mL). Data obtained from two independent experiments and represented as means ± SEM. §: below the detection limit.

To identify at which step in HCV life cycle WV can inhibit the viral replication (before, during or after viral adsorption), the following time-of-addition experiments were performed, and the results are shown in Figure 2. First, to examine if there is any possible interaction between the cells and WV, Huh7-it cells were pre-incubated with 500 ng/mL of WV for 2 h at 37 ºC before HCV adsorption. Virus production in the culture supernatants at 1 and 2 dpi were determined [Figure 2, (a) Pre-cells]. No statistically significant differences were observed with regard to virus production in the culture supernatants of WV-treated and untreated cells, indicating that WV treatment did not induce antiviral state in the venom treated cells. Then, the potential virucidal activity of the WV was evaluated, in which HCV was incubated in medium containing 500 ng/mL of WV for 2 h at 37 ºC before virus inoculation [Figure 2, (b) Pre-virus]. After 2 h, the cells were washed and re-incubated with fresh medium without WV thereafter. Under this experimental condition, the production of HCV infectious particles in culture supernatants was completely inhibited at 1 and 2 dpi. This result suggested the possibility that WV affected the virion directly thus preventing viral adsorption and entry to the cell. 

**Figure 3.**
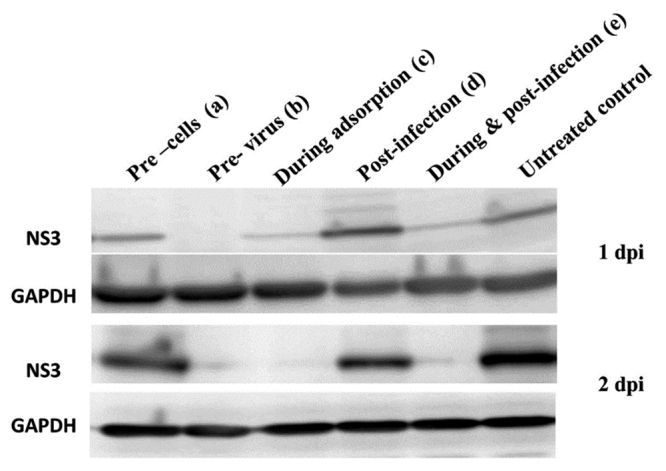
Effect of the addition of WV at various times during HCV infection on Huh7it-1 cells. JFH-1 HCV and WV (500 ng/mL) were added to Huh7it-1 cells at various time points. **(a-e)** Accumulation of HCV NS3 protein inside the cells. Virus-infected cells were analyzed by immunoblot using an anti-HCV NS3 antibody at 1 and 2 dpi. Signals of NS3 were normalized to the corresponding GAPDH signal. dpi: days post-infection.

**Figure 4.**
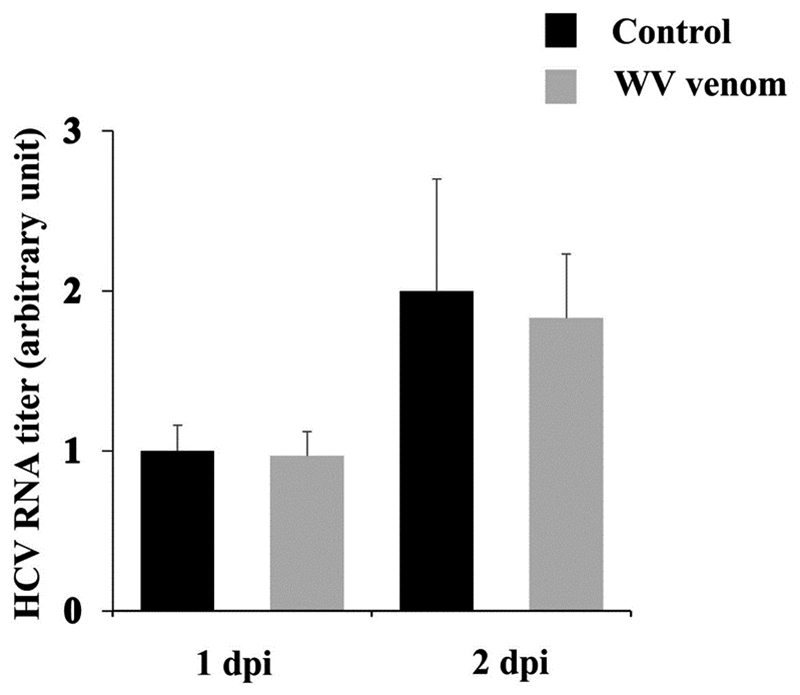
WV post-infection treatment does not affect HCV RNA levels in the infected cells. Huh7it-1 cells were infected with HCV and then treated with the venom (500 ng/mL). At 1 and 2 dpi, HCV RNA was quantified by using qRT-PCR and normalized to GAPDH mRNA expression.

Next, to check the effect of WV during the adsorption period, the venom was added to the cells during viral inoculation. After 2 h, the cells were washed and re-incubated with fresh medium thereafter [Figure 2, (c) During adsorption]. The result showed partial inhibition of HCV production in the culture; WV inhibited viral production at 1 dpi but not at 2 dpi. Finally, in order to evaluate the effect of WV after virus inoculation, HCV-infected cells were cultured in the medium containing WV (500 ng/mL) for 1 and 2 days [Figure 2, (d) Post-infection and (e) During & post-infection]. The result revealed that WV, when added to HCV-treated cells, suppressed viral infectivity in culture supernatants, suggesting that HCV infectivity outside the cells (in culture supernatants) and/or HCV replication inside the cells was inhibited. To further clarify these possibilities, we examined HCV protein synthesis (inside the cells) by immunoblot analysis (Figure 3). The results clearly demonstrated that HCV nonstructural protein 3 (NS3) synthesis was not inhibited by post-inoculation treatment while it was completely inhibited by pretreatment of virus for 2 h. Moreover, HCV RNA levels in the cells were not significantly affected by post-infection treatment (Figure 4). Taken together, these results strongly suggest that WV affects the HCV particles directly to prevent viral adsorption and entry to the cells.

## Discussion

Many of commercial antiviral drugs can cause severe and considerable side effects, particularly to those patients with chronic diseases and receiving treatment for lifelong. In addition, most of viruses have rapid mutational rate to trick host cells and infect it. For these reasons, the seeking for new antiviral drugs, especially from natural products is immediately needed. Animal venoms are important sources for natural bioactive molecules. They have by themselves a high medicinal properties to cure disease, thus it is logically to be considered as a potential source for drug discovery [17-19]. 

The Hymenoptera is one of the largest orders among insects. It is composed of at least 200,000 species of bees, wasps and ants. Most of them have stinging venom, utilized for prey capture and self-defense. Hymenoptera venom therapy was known and performed in the era of ancient Egypt, China, and Greece[20]. Three families of the order Hymenoptera; Formicidae (ants), Vespidae (yellow jackets, hornets and paper wasps commonly referred as wasps) and Apidae (bees), are medically important groups of stinging insects [21]. 

Wasps are members of the order Hymenoptera and distributed in several parts of the world. They have two different lifestyles; solitary and social. Social wasps, including hornets, use venom as a defensive measure to protect their colonies, whereas solitary wasps use their venom to capture prey. In our study, we investigated the antiviral activity of the crude venom of the Oriental hornet *V. orientalis*. The crude venom of this insect has anti-HCV activity, with IC_50_ value being 10 ng/mL with CC_50_ of 11,000 ng/mL and SI of 1,100. The results also showed that 2-hour incubation of HCV viral particles with WV suppressed the virus infectivity, most probably through a direct virucidal activity. Similarly, the same treatment completely inhibited the production of HCV infectious particles in culture supernatants at 1 and 2 dpi. 

On the other hand, we also observed that WV completely suppressed HCV infectivity in culture supernatants during post-inoculation at 1 and 2 dpi. However, high level expression of intracellular HCV protein was detected at the same time points. Possibly, during the 2 days of incubation the dose of WV (500 ng/mL) may be sufficient to neutralize newly synthesized viral particles and thus inhibited any new viral infection supporting the idea of WV virucidal activity. Thus, it is likely that WV contains a compound(s) that induces anti-HCV virucidal activity. In this regard, venom of honeybee, a social hymenopteran insect, showed 200 times more potent virucidal activity against HCV than WV did [8]. Such difference in antiviral activity may be due to the variability in venom composition between the two insect species. 

Components of wasp venom are commonly categorized as: (i) bioactive molecules such as serotonin, histamine, catecholamines, tyramine, acetylcholine, flavonoids, and biologically active amines; (ii) high molecular weight proteins such as, hyaluronidases, phospholipases, antigen 5 etc. and (iii) low molecular weight peptides such as, mastoparan, as all revealed a great potential source for drug discovery [3]. It is known that mastoparan, a wasp kinin and chemotactic peptide, is a tetra decapeptide present in wasp venom [22] in the forms of amphipathic helical structures. Mastoparan can form pores in lipid bilayers of erythrocytes, bacteria and mast cells [22]. Moreover, mastoparan structure causing pore formation and disruption to the viral lipidic envelope [23]. Thus, it is conceivable that the mastoparan in WV may be responsible for the observed virucidal activity against HCV. In this connection, a mastoparan analogue (Mastoparan-7) exhibited a wide spectrum of antiviral activity in *in vitro* assays against six different families of enveloped viruses [23]. 

Vespoid wasp phospholipases are able to disrupt the phospholipid packings of several types of biological membranes leading to pore formation and/or cell lysis through catalyzes the specific hydrolysis the ester bonds at the sn-2 position of 1,2-diacyl-3-snglycerophospholipids with release of free fatty acids [24,25]. Vespoid phospholipase A_2_ (PLA_2_) has very potent cytolytic actions, suggesting the PLA_2_ may be responsible for the WV virucidal activity against HCV. The mastoparans are also potent stimulants of purified PLA_2_ from different sources [26]. It is possible that PLA_2_ can act in synergism with mastoparan to induce virucidal activity through cytotoxin/mastoparan and PLA_2_ complex formation in wasp venom. It has been shown recently that PLA_2_ can act in synergism with melittin to induce cytotoxicity through cytotoxin/melittin and PLA_2_ complex formation [27]. 

Here, we demonstrated that WV possesses a virucidal (neutralizing) activity against HCV particles. WV may exert its antiviral effect through a common or non-common molecule(s) or toxin complex within the crude venom. Further studies for characterization and identification of active compound(s) responsible for the antiviral activity of WV are needed.

## Conclusion

Throughout this study, we found that WV can inhibit the entry stage of HCV infection at non-cytotoxic concentrations with no inhibitory effects on replication of HCV. Therefore, it can be considered a good candidate for characterization of new and natural anti-HCV agents targeting the entry step. The exact mechanism by which WV exerts its antiviral activity against HCV to inhibit infecting the target cells still requires further studies. 

**Abbreviations**

CC50: 50% cytotoxic concentration; DAAs: direct-acting antivirals; dpi: days post-infection; FDA: Food and Drug Administration; GAPDH: human glyceraldehyde 3-phosphate dehydrogenase; HCC; hepatocellular carcinoma; HCV: hepatitis C virus; HRP: horse-radish peroxidase; Huh7it-1: human hepatocellular carcinoma-derived cell line; IC50: 50% inhibitory concentration; JFH1: recombinant hepatitis C virus J6; NS3: nonstructural protein 3; PLA_2_: phospholipase A_2_; RT-qPCR: reverse transcription-quantitative polymerase chain reaction; SDS-PAGE: SDS-polyacrylamide gel electrophoresis; SEM: standard error of the mean; SI: selectivity index; SPSS: statistical package for the social sciences; WST-1: water soluble tetrazolium salts; WV: wasp venom.
